# Delta-radiomics based on CT predicts pathologic complete response in ESCC treated with neoadjuvant immunochemotherapy and surgery

**DOI:** 10.3389/fonc.2023.1131883

**Published:** 2023-05-12

**Authors:** Kaiyuan Li, Yuetong Li, Zhulin Wang, Chunyao Huang, Shaowu Sun, Xu Liu, Wenbo Fan, Guoqing Zhang, Xiangnan Li

**Affiliations:** ^1^ Department of Thoracic Surgery, First Affiliated Hospital of Zhengzhou University, Zhengzhou, Henan, China; ^2^ Clinical Medical College, Henan University, Henan, Kaifeng, China

**Keywords:** esophageal cancer, delta radiomics, neoadjuvant immunochemotherapy, pathological complete response, machine learning

## Abstract

**Background and purpose:**

Unnecessary surgery can be avoided, and more appropriate treatment plans can be developed for patients if the efficacy of neoadjuvant immunochemotherapy for esophageal cancer (EC) can be predicted before surgery. The purpose of this study was to evaluate the ability of machine learning models based on delta features of immunochemotherapy CT images to predict the efficacy of neoadjuvant immunochemotherapy in patients with esophageal squamous cell carcinoma (ESCC) compared with machine learning models based solely on postimmunochemotherapy CT images.

**Materials and methods:**

A total of 95 patients were enrolled in our study and randomly divided into a training group (n = 66) and test group (n = 29). We extracted preimmunochemotherapy radiomics features from preimmunochemotherapy enhanced CT images in the preimmunochemotherapy group (pregroup) and postimmunochemotherapy radiomics features from postimmunochemotherapy enhanced CT images in the postimmunochemotherapy group (postgroup). We then subtracted the preimmunochemotherapy features from the postimmunochemotherapy features and obtained a series of new radiomics features that were included in the delta group. The reduction and screening of radiomics features were carried out by using the Mann-Whitney U test and LASSO regression. Five pairwise machine learning models were established, the performance of which was evaluated by receiver operating characteristic (ROC) curve and decision curve analyses.

**Results:**

The radiomics signature of the postgroup was composed of 6 radiomics features; that of the delta-group was composed of 8 radiomics features. The area under the ROC curve (AUC) of the machine learning model with the best efficacy was 0.824 (0.706-0.917) in the postgroup and 0.848 (0.765-0.917) in the delta group. The decision curve showed that our machine learning models had good predictive performance. The delta group performed better than the postgroup for each corresponding machine learning model.

**Conclusion:**

We established machine learning models that have good predictive efficacy and can provide certain reference values for clinical treatment decision-making. Our machine learning models based on delta imaging features performed better than those based on single time-stage postimmunochemotherapy imaging features.

## 1 Introduction

Esophageal cancer (EC) is the most common malignant tumor of the upper digestive tract, ranking seventh in terms of incidence (604,000 new cases) and sixth in terms of overall mortality (544,000 deaths) among all cancers. The five-year relative survival rate for EC is lowest among cancers and comparable to that for liver cancer at 20% (1, 2). Most cases of EC are diagnosed at middle and advanced stages. Surgical resection after neoadjuvant chemoradiotherapy (NCRT) should be considered the standard of care for patients with resectable locally advanced EC. In patients with locally advanced ESCC, NCRT plus surgery improves survival compared with surgery alone, and the adverse events are acceptable and controllable. In patients with resectable EC, the combination of neoadjuvant chemoradiotherapy and surgery has an overall survival benefit (3, 4). Studies have reported a probability of pathologic complete response after neoadjuvant immunochemotherapy for EC of 26% to 49% (5–7). After many explorations in recent years, immunotherapy for EC has been expanded to neoadjuvant therapy and immunotherapy combined with neoadjuvant chemotherapy. There is much clinical experience accumulated to date. For example, the NICE-2, TD-NICE and Keep-G 03 studies, among others, have shown relatively ideal disease control rates and PCR rates (8–10). Therefore, it is very important to accurately predict the efficacy of neoadjuvant immunochemotherapy for EC. Radiomics is an emerging technology with the ability to capture intratumor heterogeneity in a noninvasive manner. Indeed, radiomics is a promising approach to comprehensively quantify tumor phenotypes through application of a large number of quantitative imaging features (11, 12).

Delta-radiomics is the change in radiomics features, a multitemporal comparison, and can fully reflect characteristic changes in tumors before and after treatment. Previously published studies have demonstrated the superiority of delta-radiomics in cancer from many aspects. The study of Jing Gong et al. showed that a delta-radiomics model can improve predictive performance and has prognostic value in predicting the progression-free survival and overall survival of non-small cell lung cancer (NSCLC) patients (13). According to Zhang, Z. et al., the delta-radiomics features extracted from MR images after surgical radiotherapy for brain metastases have the potential to distinguish radiation necrosis from tumor progression and have better predictive value than traditional radiomics features (14). The purpose of this study was to evaluate the ability of machine learning models based on delta features of immunochemotherapy CT images to predict the efficacy of neoadjuvant immunochemotherapy in patients with esophageal squamous cell carcinoma (ESCC) compared with machine learning models based solely on postimmunochemotherapy CT images.

## 2 Materials and methods

### 2.1 Study design

**Figure 1:**
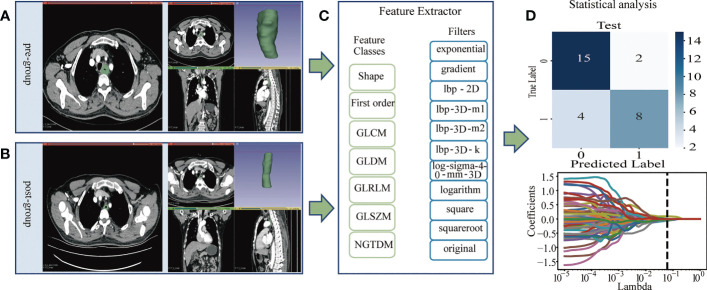
**(A, B)**: Image Acquisition & Tumor Segmentation. **(A)** The region of interest (ROI) of the pregroup. **(B)** The region of interest (ROI) of the postgroup. **(C)** The PyRadiomics package was used to extract radiomics features, and the radiomics classes and filters used are listed. The pregroup features and postgroup features were extracted, and the delta features were obtained. Delta features = postgroup features - pregroup features. **(D)** The least absolute shrinkage and selection operator (LASSO) algorithm was applied to select features, and other statistical analyses were performed, such as predictive model construction and validation.

After patient selection, we manually outlined the region of interest (ROI) on the CT images of patients before and after neoadjuvant immunochemotherapy. Then, we extracted the radiomics features according to the radiomics classes and filters shown in **Figure 1** for statistical analysis. The flowchart of the main steps is illustrated in **Figure 1**.

### 2.2 Patients

A total of 146 patients with EC who received neoadjuvant immunochemotherapy plus surgical resection at the First Affiliated Hospital of Zhengzhou University from June 2019 to May 2022 were included in this study. The inclusion criteria were (i) ESCC diagnosed by histopathology and (ii) complete available enhanced CT images before and after immunochemotherapy. The exclusion criteria were as follows: (i) adenocarcinoma of the esophagogastric junction (n=6); (ii) esophageal fistula after neoadjuvant immunochemotherapy (n=1); (iii) incomplete diagnosis and treatment process in our hospital (n=20); and (iv) nonenhanced CT or CT image artifacts (n=24). Ultimately, 95 patients were enrolled in this study.

### 2.3 CT image acquisition and tumor segmentation

Enhanced CT images before and after neoadjuvant immunochemotherapy were obtained for all patients. CT scanners from multiple manufacturers were used for enhanced chest CT of all patients. Information about the scan parameters of CT (including manufacturer, tube voltage, tube current, etc.) is provided in **Supplemental Material Table S2**. The tumor ROI was manually delineated on 3Dslicer (version 4.1.1, http://www.slicer.org, USA) by two thoracic surgeons with more than 5 years of clinical experience; the ROI was delineated and analyzed in the arterial phase. First, the tumor contour was delineated on enhanced CT images before neoadjuvant immunochemotherapy by referring to gastroscopy, barium meal gastrointestinal examination and other examinations. Then, the head and tail lengths of the delineated ROI on the enhanced CT images before and after neoadjuvant immunochemotherapy were determined. The length was kept unchanged, and the tumor ROI after neoadjuvant immunochemotherapy continued to be delineated manually. CT images of the same patient before and after neoadjuvant immunochemotherapy ensured that the tumor was of the same length in the sagittal position. The preneoadjuvant target area served as a reference for the postneoadjuvant target area; that is, the target area was the same. The mapped tumor area was evaluated by another radiologist.

### 2.4 Radiomics feature extraction and selection

The PyRadiomics (version 3.0.1, http://github.com/Radiomics/pyradiomics#readme) package of Python software (version 3.9.7) was used to extract features from the postgroup and delta group. Delta radiomics features were defined as the radiomics features of the postgroup minus the radiomics features of the pregroup. The extracted radiomics features were screened by the Mann-Whitney U test, and features with a threshold of P < 0.05 were retained, after which data standardization (StandardScaler) was selected to nondimensionalize these retained radiomics features. Next, five cross-validations and iterations of 1e6 were performed on the standardized features to obtain the alpha parameter with the minimum mean square error. Based on the selected optimal alpha parameter, the least absolute contraction and selection operator (LASSO) feature selection algorithm was applied to select relevant features and calculate the coefficients of each. LASSO solves the multicollinearity problem by resetting insignificant feature weights to zero through penalty coefficients, thus reducing the feature dimension. Finally, radiomics features with nonzero coefficients were obtained. To increase the repeatability of radiomics features and the generalization and stability of the models, 10 patients in the pregroup and the postgroup were randomly selected, and the ROIs outlined by Reader1 and Reader2 were used for reliability analysis (ICC). Detailed information is available in **Supplementary Materials Table S3** and **Table S4**.

### 2.5 Statistical analysis

We randomly divided patients into a training set and a test set (7:3); the former was used to develop the machine learning models and the latter to verify and evaluate the performance of the machine learning models. The predictive radiomics features selected by the Mann-Whitney U test and LASSO algorithm were entered into machine learning models. We built the machine learning models using the scikit-learn package (version 1.0.2, http://scikit-learn.org) in Python (version 3.9.7). Five machine learning models were constructed with both the postgroup and delta group, including support vector machine (SVM), regression decision tree (DT), random forest (RF), extreme gradient boosting (XGBoost), and logistic regression (LR). We also evaluated the predictive power of each machine learning classifier using a validation set, and the AUC value and the corresponding sensitivity, specificity, and overall accuracy were calculated. Decision curves of the machine learning models with the best AUC performance were plotted for guiding clinical decisions.

## 3 Results

### 3.1 Patients

A total of 95 patients were enrolled in our study. Postoperative histopathologic specimens were evaluated by an experienced pathologist and reviewed by a thoracic surgeon. Pathological complete response (PCR) occurred in 39 patients and nonpathological complete response (nPCR) in 56 patients, with a ratio of approximately 2:3. **Table 1** provided the patient details. The immunochemotherapy regimen was paclitaxel and platinum combined with PD-1 monoclonal antibody. Ninety-four patients underwent esophagectomy by the McKeown method, and 1 patient underwent esophagectomy by the Ivor-Lewis method.

**Table 1:** Demographic statistics of patients in the training cohort and test cohort.

Variable	Training cohort (n=66)	Test cohort(n=29)		
			χ2/Z	P
**Sex**			0.4107	0.5216
** Female**	25	9		
** Male**	41	20		
**Age**			-0.931	0.352
** Mean**	65.3	64.1		
** Median**	66	64		
** Range**	49~77	54~75		
** SD**	6.39	6.12		
**Smoking history**			0.028	0.8671
** Yes**	17	7		
** No**	49	22		
**Alcohol history**			2.0285	0.1544
** Yes**	10	8		
** No**	56	21		
**BMI**			0.3177	0.573
** ≥ 18.5 and <24**	36	14		
** ≥ 24**	30	15		
**Clinical T stage**			0.325	0.9553
** T1**	17	7		
** T2**	14	5		
** T3**	26	13		
** T4**	9	4		
**Clinical N stage**			5.9933	0.05
** N0**	46	13		
** N1**	14	13		
** N2**	6	3		
**Tumor location**			2.7979	0.2469
** Upper thoracic**	10	1		
** Middle thoracic**	32	17		
** Lower thoracic**	24	11		
**Pathological Differentiation**			2.9638	0.3972
** Low**	7	7		
** Middle**	31	12		
** High**	3	1		
** unknown**	25	9		

### 3.2 Feature selection of radiomics

**Figure 2:**
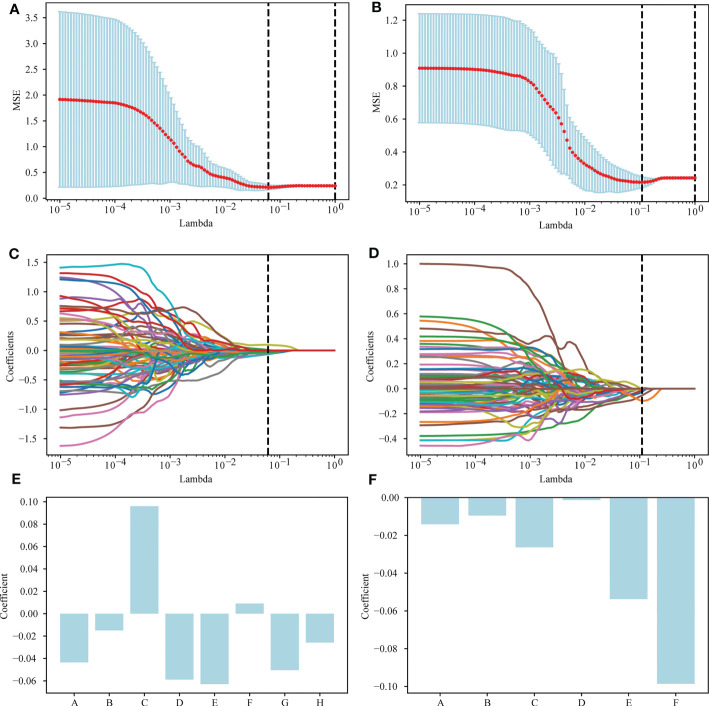
Selection of radiomics features *via* the LASSO method. **(A)** A 5-fold cross-validation curve for the radiomics features of the delta group, with vertical dashed lines drawn at the point where the optimal lambda value is 0.0613591 and the number of radiomics features is 8. **(B)** A 5-fold cross-validation curve for the radiomics features of the postgroup was drawn with vertical dashed lines at the optimal lambda value of 0.1097498 and the number of radiomic features of 6. **(C)** LASSO coefficient profiles of the 154 features retained for the delta group. The coefficient profile is drawn for the lambda sequence. Vertical lines are drawn at values selected using 5-fold cross-validation, where the optimum lambda yields 8 features with nonzero coefficients. **(D)** LASSO coefficient profiles of the 335 features retained for the postgroup. The coefficient profile is drawn for the lambda sequence. Vertical lines are drawn at values selected using 5-fold cross-validation, where the best lambda yields 6 features with nonzero coefficients. **(E, F)** The nonzero coefficients screened by post group and their corresponding coefficients.

A total of 1,037 features were extracted from the postgroup and the delta group. The extracted radiomics features were screened by the Mann-Whitney U test, and those with a threshold of P < 0.05 were retained. In total, 335 features for the postgroup were retained and 154 for the delta group. Then, the least absolute shrinkage and selection operator (LASSO) algorithm was applied to select features. Details are provided in **Table S1** in the **Supplementary Material**. Among them, 6 nonzero features were retained for the postgroup and 8 for the delta group, and their corresponding coefficients were determined. For the postgroup, the results were as follows: coefficient of feature A ‘original_shape_Maximum2DDiameterSlice’ -0.01413; of feature B ‘lbp-2D_gldm_DependenceNonUniformityNormalized’ -0.00953215; of feature C ‘lbp-3D-m2_firstorder_Kurtosis’ -0.0263521; of feature D ‘lbp-3D-m2_gldm_DependenceNonUniformityNormalized’ -0.00134778; of feature E ‘lbp-3D-k_glszm_SmallAreaLowGrayLevelEmphasis’ -0.05375544; and of feature F’square_glrlm_RunEntropy’ -0.09855901. The results for the postgroup the delta group were as follows: coefficient of feature A ‘original_shape_Elongation’ -0.04356936; of feature B ‘original_shape_MinorAxisLength’ -0.1491607; feature C ‘original_shape_SurfaceVolumeRatio’ 0.09608021; of feature D ‘lbp-3D-m2_firstorder_Kurtosis’ -0.05886248; of feature E ‘lbp-3D-k_glszm_SmallAreaHighGrayLevelEmphasis’ -0.06292696; of feature F ‘lbp-3D-k_ngtdm_Coarseness’ 0.0091555; of feature G ‘log-sigma-4-0-mm-3D_glcm_Autocorrelation’ -0.05041306; and of feature H’square_glrlm_RunEntropy’ of -0.02573776. These results are shown in **Figure 2**.

### 3.3 Diagnostic performance of radiomics models

The nonzero features of the two groups retained were modeled separately by machine learning, and all models showed good predictive efficacy of neoadjuvant immunochemotherapy in the validation set. The results for all models are given in **Table 2**.

**Table 2:** The results of all models.

Models	post						delta					
	SEN	SPE	PPV	NPV	ACC	AUC	SEN	SPE	PPV	NPV	ACC	AUC
**SVM**	0.58	0.71	0.58	0.71	0.66	0.686(0.667-0.706)	0.58	0.88	0.78	0.75	0.76	0.770(0.667-0.882)
**DT**	0.58	0.76	0.64	0.72	0.69	0.711(0.706-0.750)	0.67	0.82	0.73	0.78	0.76	0.745(0.667-0.824)
**RF**	0.83	0.76	0.71	0.87	0.79	0.824(0.706-0.917)	0.67	0.88	0.8	0.79	0.79	0.848(0.765-0.917)
**XGBoost**	0.33	0.82	0.57	0.64	0.62	0.760(0.647-0.833)	0.58	0.88	0.78	0.75	0.76	0.789(0.647-0.833)
**LR**	0.58	0.71	0.58	0.71	0.66	0.676(0.647-0.750)	0.67	0.71	0.62	0.75	0.69	0.799(0.667-0.824)

**Figure 3:**
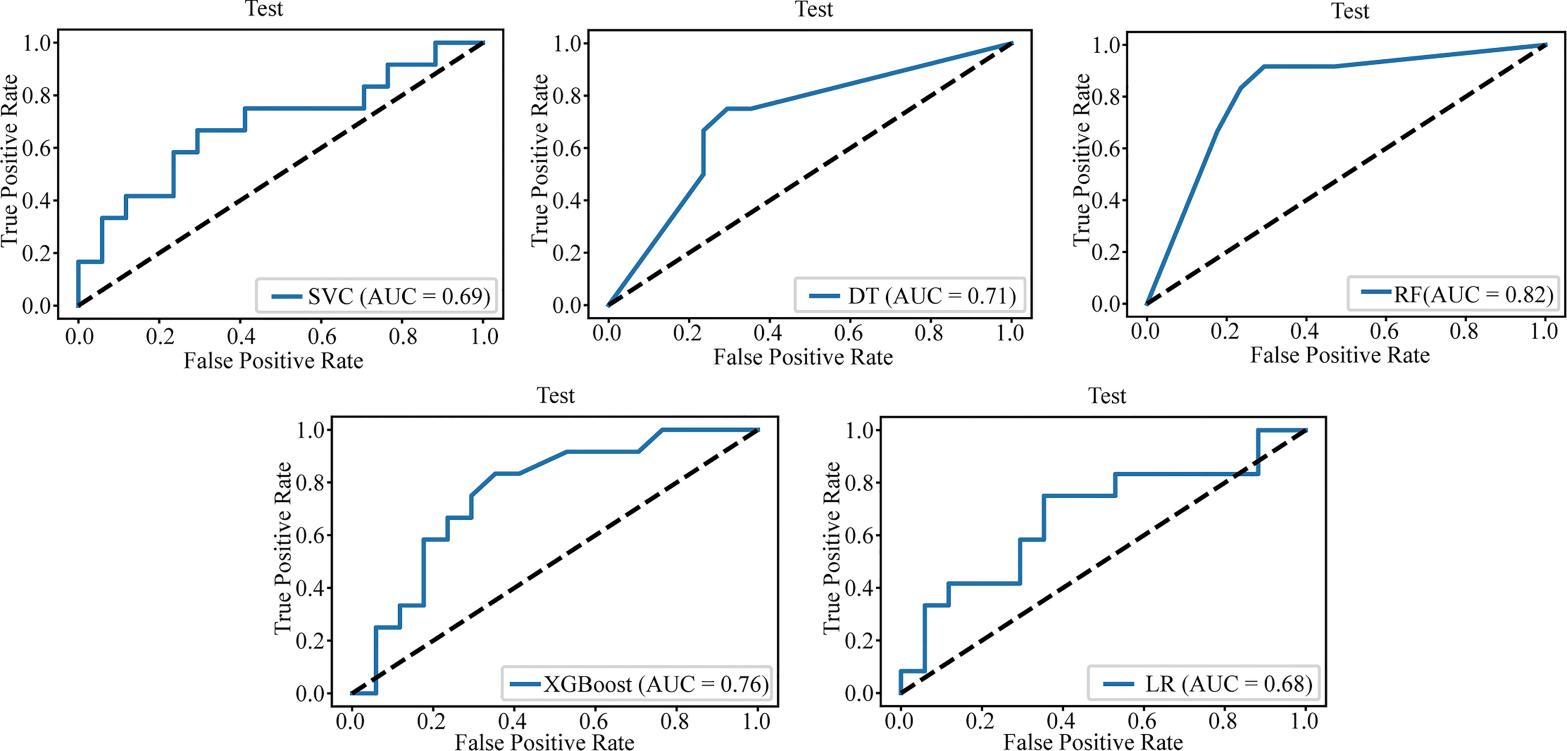
The AUC value for the post group test set.

**Figure 4:**
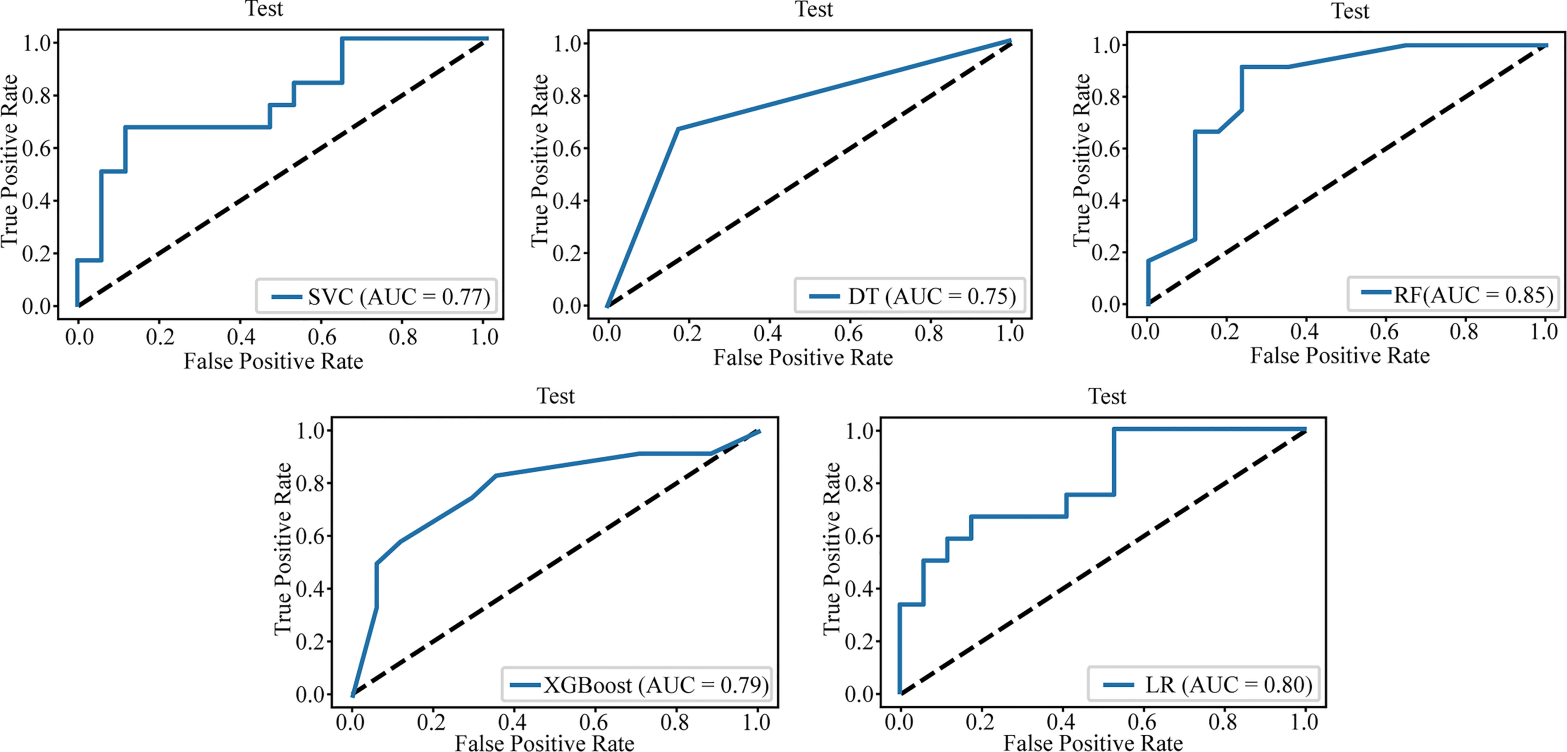
The AUC value for the delta group test set.

**Figure 5:**
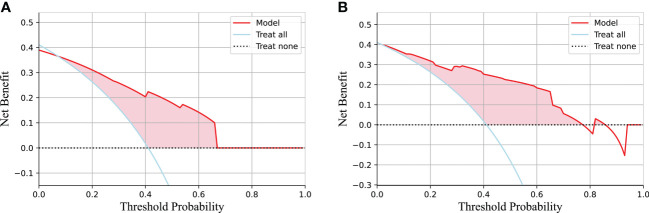
The decision curve of the random forest model based on the test group and two extreme curves are drawn. The decision curve depicts the net benefit of the model within a certain probability threshold (Y-axis). The treat-all curve indicates that the intervention was performed regardless of the predicted outcome; thus, esophagectomy was avoided in this study. The treat-none curve indicates that no intervention was performed regardless of the outcome; thus, esophagectomy was performed in this study. The part of the model that is better than the two extreme curves is indicated by the pink fill. **(A)** The decision curve for the postgroup. **(B)** The decision curve for the delta group.

**Table 3** lists the specific parameters of each model. In the process of model fitting, we use grid searches, learning curves and other methods to obtain optimal parameters. The random forest classifier had the best effect in both groups. The AUC value of the validation set of the postgroup was 0.82 (95% CI, 0.706-0.917); the sensitivity was 0.83, the specificity 0.76, and the accuracy 0.79. The AUC value of the delta group was 0.85 (95% CI, 0.765-0.917), the sensitivity was 0.67, the specificity was 0.88, and the accuracy was 0.79. **Figures 3**, **4** depict all the ROC curves of the models. In this study, we plotted decision curves of the best-performing random forest classifiers to guide clinical decision-making, as indicated in **Figure 5**. Decision curve analysis (DCA) is a widely used method to measure clinical practicability. **Figure 5** shows the net benefit of two random forest models in determining the efficacy of immunochemotherapy for ESCC. The net benefit was defined as the harm from a residual tumor by avoiding surgical resection of the esophagus (false positive) subtracted from the benefit from avoiding surgical resection of the esophagus (true positive) in patients predicted by the model to have PCR. It can be seen from the decision curve that the random forest model of the delta group indicated more net benefit than the random forest model of the postgroup.

**Table 3:** Parameters of all machine learning models in this study.

groups	Model	parameters
**delta**	SVM	C = 25, gamma = 0.09, kernel = “poly”, probability = True
	DT	criterion = ‘entropy’, random_state = 23, max_depth = 1, min_samples_leaf = 1, min_samples_split = 5
	RF	random_state = 11, n_estimators = 3, criterion = “gini”, max_depth = 2, max_features = “sqrt”, min_samples_leaf = 1, min_samples_split = 5
	XGBoost	random_state = 10, booster = ‘gbtree’,learning_rate=0.23,colsample_bylevel=0.8,gamma =0,max_depth=3,min_child_weight=3,n_estimators = 3, objective=‘binary:logistic’,use_label_encoder=False
	LR	penalty=“l1”, solver=“liblinear”,C = 0.1125,class_weight=“balanced”
**post**	SVM	C = 0.0625, gamma = 0.01, kernel = “linear”, probability = True
	DT	criterion = ‘entropy’, random_state = 21, max_depth = 6, min_samples_leaf = 2, min_samples_split = 7
	RF	random_state = 26, n_estimators = 2, criterion = “gini”, max_depth = 2, max_features = “auto”, min_samples_leaf = 1, min_samples_split = 5
	XGBoost	random_state = 10, booster = ‘gbtree’,learning_rate=0.55,colsample_bytree = 0.1, colsample_bylevel=0.6,gamma =0,max_depth=3,min_child_weight=3,n_estimators = 5, objective=‘binary:logistic’,use_label_encoder=False
	LR	penalty=“l1”, solver=“liblinear”,C = 0.3,class_weight=“balanced”,tol=0.0001, multi_class=‘ovr’

## 4 Discussion

The purpose of this study was to evaluate the difference in the prediction of neoadjuvant immunochemotherapy for ESCC between postimmunohistotherapy CT modeling alone and delta imaging modeling. We used the variation in image group characteristics before and after immunochemotherapy (delta group) and the image of the individual postgroup to build 5 kinds of machine learning models, which were verified in the test set. Each machine learning model showed good predictive ability with regard to the effect of a neoadjuvant immunochemotherapy curative effect, and the prediction effect was best in the random forest models. Indeed, the two random forest models achieved high AUC values of 0.82 (postgroup) and 0.85 (delta-group) in the verification set. The predictive effect of the model established by delta radiomics was better than that of single imaging feature modeling after immunochemotherapy. The AUC value was similar to that reported by Hu, Y., et al. (15) Moreover, the results were verified by DCA, demonstrating good clinical practicability of the models.

Traditional imaging examination can show the size, morphology, enhancement mode and other characteristics of lesions but cannot reveal more in-depth information about EC. As an emerging technology to capture high-throughput imaging features, radiomics can capture the heterogeneity of tumors in a noninvasive way with great objectivity. Many previous studies have demonstrated the utility of radiomics in predicting response to neoadjuvant chemoradiotherapy at different levels. Most of these studies used FDG PET/CT to predict PCR with neoadjuvant chemotherapy and radiotherapy (NCRT) in patients with locally advanced EC (16–18). In addition, most previous studies modeled radiomics features based on a single time phase. In the study of Qiu Q et al., CT images of patients before nCRT were collected, 711 radiomics features were extracted, and radiomics nomograms were constructed. The optimal value of the C index was 0.746 (95% CI, 0.680 – 0.812) in the training cohort and 0.724 (95% CI, 0.696 – 0.752) in the validation cohort (19). Mao, Y. et al. also extracted a total of 340 radiological features from CT images of patients with locally advanced rectal cancer (LARC) prior to neoadjuvant chemotherapy. The best performing model used both radiomics and clinical variables, with areas under the curve of 0.926 and 0.872, respectively, in the training and validation cohorts (20). Similarly, Yang Z et al. extracted radiomic features from CT images before neoadjuvant therapy and constructed three models. The AUC values of the model with the best performance in the training set and the test set were 0.85 and 0.79, respectively (21). However, studies based on a single phase did not contain information about response to treatment. Delta radiomics covers a large amount of time-dependent information, allows dynamic assessment of complete tumor changes over the treatment period, provides a large amount of data on treatment-induced changes and is more consistent with assessment of immunotherapy effects in clinical practice. Thus far, few previous studies have used delta imaging features to model and predict the efficacy of neoadjuvant chemotherapy for EC. Xie CY et al. used a delta radiomics approach combined with a genomics approach that utilized differentially expressed genes to reduce the number of radiomics features, allowing the creation of a CT-based radiomics model using a genomic-based feature selection approach. This resulted in better performance and versatility (AUC: 0.912 in the training set, 0.825 in the internal test set, and 0.749 in the external test set) (22). In recent years, the unique value of delta radiomics has been demonstrated in many areas of cancer and shown to improve the performance of predictive models in many ways (23–28). This is the same as the conclusion obtained in this study, which is encouraging. In this study, comparison between radiomics models was conducted based on CT images for the same patients, and the results showed that delta radiomic features were superior to single time-phase image omics features, which may provide certain reference value for similar radiomics modeling in the future.

This study built a decision curve based on the best machine learning model, with potential clinical application for some problems based on machine learning model and decision curve analyses. EC patients have a high incidence of surgical complications, significantly reduced postoperative quality of life and risk of death, which is not a good choice for patients who achieve PCR after neoadjuvant chemotherapy (29–32). The results of this study can be used as a potential auxiliary method independent to evaluate surgical specimens to identify complete responders who may avoid surgery and as an important reference factor to evaluate whether patients can undergo neoadjuvant chemotherapy as an alternative therapy to surgery. This approach provides significant clinical benefit for identifying patients eligible for individualized organ preservation therapy programs (33).

There are some limitations in this study. The main limitation is that the sample size was small. The main reason for this is that complete patient information was involved, complete and available enhanced CT images before and after neoadjuvant chemotherapy had to be available, and postoperative pathological confirmation and accuracy of follow-up information had to be completed. In addition, to ensure the learning effect of machine learning, we conducted data balancing (PCR: none-PCR = 1:1), as the aforementioned findings suggested that the probability of pathologic complete response after neoadjuvant chemotherapy for esophageal cancer is 26% to 49% (5–7). Therefore, the clinical PCR rate also limited the sample size. This may have resulted in a model that was weak in generalizability and does not represent the characteristics of all populations. Second, more medical centers were needed, and it would be worthwhile to conduct research involving more centers. Multicenter research may be helpful to improve and externally verify our machine learning model, increase its ability to assist in therapy, and increase its ability to contribute to clinical decision-making and effective prediction.

## 5 Conclusion

We used CT to extract radiomics features to establish a sample machine learning model for effectively predicting PCR after neoadjuvant immunochemotherapy. The machine learning model we established has a good predictive effect and can provide some value for clinical treatment decision-making. Overall, our machine learning model based on delta imaging features performed better than the model based on single time-phase postimmunochemotherapy imaging features.

## Data availability statement

The original contributions presented in the study are not publicly available, but are available from the corresponding author on reasonable request.

## Ethics statement

Written informed consent was obtained from the individual(s) for the publication of any potentially identifiable images or data included in this article.

## Author contributions

KL and YL designed the experiments, performed the study, completed the data analysis and wrote the first draft of the paper. CH, ZW, SS, XuL, and WF participated in the experimental design and analysis of the experimental results. GZ and XiL conceived of the idea for the project and is the author and person in charge guided the experimental design, data analysis, and manuscript writing and revision. All authors contributed to the article and approved the submitted version.
