# Supplementary material for: Delta-radiomics based on CT predicts pathologic complete response in ESCC treated with neoadjuvant immunochemotherapy and surgery
Source: Front Oncol. 2023 May 12;13:1131883. doi: 10.3389/fonc.2023.1131883 (PMC10213404; doi:10.3389/fonc.2023.1131883)
Supplement: Supplementary file 1 [file DataSheet_1.docx]

Supplementary Material

Delta-Radiomics Based on CT Predicts Pathologic Complete Response in ESCC Treated with Neoadjuvant Immunochemotherapy and Surgery

Kaiyuan Li^1^†, Yuetong Li^2^†, Zhulin Wang^1^, Chunyao Huang^1^, Shaowu Sun^1^, Xu Liu^1^ and Wenbo Fan^1^, Guoqing Zhang ^1*^, Xiangnan Li^1*^

†These authors contributed equally to this work and share first authorship

^1^ Department of Thoracic Surgery, First Affiliated Hospital of Zhengzhou University, Zhengzhou 450052, Henan Province, China

^2^Student majoring in clinical medicine, Henan University, Henan Province, China

*** Correspondence:**Xiangnan Li
[lxn-2000@163.com](mailto:lxn-2000@163.com)

Guoqing Zhang

drzhangguoqing@163.com

# Supplementary Figures and Tables

Table S1: All the radiomics features extracted and the screening process.

| Feature Classes | Features | original | Filters | | | | | | | | | |
| --- | --- | --- | --- | --- | --- | --- | --- | --- | --- | --- | --- | --- |
|  |  |  | exponential | gradient | lbp-2D | lbp-3D-m1 | lbp-3D-m2 | lbp-3D-k | log-sigma-4-0-mm-3D | logarithm | square | squareroot |
| shape | Elongation | d/**△^#^** | NONE^*^ | | | | | | | | | |
|  | Flatness | d**^#^** |  |  |  |  |  |  |  |  |  |  |
|  | LeastAxis  Length | p/d |  |  |  |  |  |  |  |  |  |  |
|  | MajorAxis  Length |  |  |  |  |  |  |  |  |  |  |  |
|  | Maximum2DDiameterColumn |  |  |  |  |  |  |  |  |  |  |  |
|  | Maximum2DDiameterRow |  |  |  |  |  |  |  |  |  |  |  |
|  | Maximum2DDiameterSlice | p/d/**√** |  |  |  |  |  |  |  |  |  |  |
|  | Maximum3DDiameter | p**^#^** |  |  |  |  |  |  |  |  |  |  |
|  | MeshVolume | d |  |  |  |  |  |  |  |  |  |  |
|  | MinorAxisLength | p/d/**△** |  |  |  |  |  |  |  |  |  |  |
|  | Sphericity | p/d |  |  |  |  |  |  |  |  |  |  |
|  | SurfaceArea | d |  |  |  |  |  |  |  |  |  |  |
|  | SurfaceVolumeRatio | p/d/**△** |  |  |  |  |  |  |  |  |  |  |
|  | VoxelVolume | p/d |  |  |  |  |  |  |  |  |  |  |
| firstorder | 10Percentile | p | p |  |  | p/d | p |  |  |  |  | p |
|  | 90Percentile |  |  |  |  | p/d | p |  |  |  | p |  |
|  | Energy | p |  | p | p | p | p | p | p | p | p | p |
|  | Entropy | p |  |  |  |  |  |  | p |  |  |  |
|  | InterquartileRange |  |  |  |  |  | p/d |  | p |  |  |  |
|  | Kurtosis |  |  |  | d | p/d | p/d/**△**/**√^#^** |  |  |  |  |  |
|  | Maximum |  |  |  |  |  |  | p | p/d |  |  |  |
|  | MeanAbsoluteDeviation | p |  |  | d | p/d | p/d |  | p |  | p |  |
|  | Mean |  |  |  | p/d |  |  |  |  |  | p |  |
|  | Median |  |  |  |  |  |  |  |  |  |  |  |
|  | Minimum | p | p |  |  |  |  |  | d |  |  |  |
|  | Range | p |  |  |  |  |  | p | p/d |  |  |  |
|  | RobustMeanAbsoluteDeviation |  |  |  | d | p/d | p/d |  | p |  |  |  |
|  | RootMeanSquared | p |  |  | d | p/d |  | p/d | p |  | p |  |
|  | Skewness |  |  |  | d |  |  | p | p |  |  |  |
|  | TotalEnergy | p | d | p | p | p/d | p/d | p/d | p |  | p | p |
|  | Uniformity | p |  |  |  |  |  |  | p |  |  |  |
|  | Variance | p |  |  | d | p/d | p |  | p |  | p |  |
| GLCM | Autocorrelation |  |  |  |  |  |  |  | p/d/**△** |  | p |  |
|  | ClusterProminence | p |  |  |  |  |  |  | p |  | p |  |
|  | ClusterShade |  |  |  |  |  |  |  | p |  | p |  |
|  | ClusterTendency | p |  |  |  |  |  |  | p |  | p |  |
|  | Contrast |  |  |  |  |  |  |  | p |  |  |  |
|  | Correlation |  |  | p |  |  |  | p |  |  |  |  |
|  | DifferenceAverage |  |  |  |  |  |  |  | p |  |  |  |
|  | DifferenceEntropy |  |  |  |  |  |  |  | p |  |  |  |
|  | DifferenceVariance |  |  |  |  |  |  |  | p/d |  |  |  |
|  | Id |  |  |  |  |  |  |  |  |  |  |  |
|  | Idm |  |  |  |  |  |  |  |  |  |  |  |
|  | Idmn |  |  |  |  |  |  |  |  |  |  |  |
|  | Idn |  |  |  |  |  |  |  |  |  |  |  |
|  | Imc1 |  |  |  |  |  |  |  | d |  | p |  |
|  | Imc2 |  |  |  |  |  |  |  |  |  | p |  |
|  | InverseVariance |  |  |  |  |  |  |  |  |  |  |  |
|  | JointAverage |  |  |  |  |  |  |  | p/d |  | p |  |
|  | JointEnergy | p |  |  |  |  |  |  | p |  |  |  |
|  | JointEntropy |  |  |  |  |  |  |  | p |  |  |  |
|  | MCC |  |  |  |  |  |  |  |  |  |  |  |
|  | MaximumProbability |  |  |  |  |  |  |  | p |  |  |  |
|  | SumAverage |  |  |  |  |  |  |  | p/d |  | p |  |
|  | SumEntropy | p |  |  |  |  |  |  | p |  |  |  |
|  | SumSquares | p |  |  |  |  |  |  | p |  | p |  |
| GLDM | DependenceEntropy | p/d | p/d | p | p/d | p/d | p/d |  | p |  |  | p |
|  | DependenceNonUniformity | p | p | p | p | p | p | p | p | p | p | p |
|  | DependenceNonUniformityNormalized |  | p/d |  | p/d/**√** | p/d | p/d/**√** |  |  |  |  |  |
|  | DependenceVariance |  | p/d |  | p/d | p/d | p/d |  |  |  |  |  |
|  | GrayLevelNonUniformity |  | p |  | p | p | p | p |  |  |  |  |
|  | GrayLevelVariance | p |  |  |  |  |  |  | p |  | p |  |
|  | HighGrayLevelEmphasis |  |  |  | p |  |  |  | p/d |  | p |  |
|  | LargeDependenceEmphasis |  | p/d |  | d | p/d | p/d |  |  |  |  |  |
|  | LargeDependenceHighGrayLevelEmphasis |  | p/d | p | p/d | p/d | p/d | p | d |  | p |  |
|  | LargeDependenceLowGrayLevelEmphasis |  | p/d |  | p/d | p/d | p/d |  | d | p |  | p |
|  | LowGrayLevelEmphasis |  |  |  | p |  |  |  | p/d | p |  | p |
|  | SmallDependenceEmphasis |  | p/d |  | d | p/d | p/d | p/d |  |  |  |  |
|  | SmallDependenceHighGrayLevelEmphasis |  | p/d |  | p/d | p/d | p/d | p/d | p/d |  |  |  |
|  | SmallDependenceLowGrayLevelEmphasis |  | p/d |  | p/d | p/d | p/d | p/d | d |  |  |  |
| GLRLM | GrayLevelNonUniformity |  |  | p |  |  |  | p |  |  |  |  |
|  | GrayLevelNonUniformityNormalized | p |  |  |  |  |  | p/d | p |  | p |  |
|  | GrayLevelVariance | p |  |  |  |  |  | p/d | p |  | p |  |
|  | HighGrayLevelRunEmphasis | p/d |  |  |  |  |  | p | p/d |  | p |  |
|  | LongRunEmphasis |  | p |  | p | p | p | p |  |  |  |  |
|  | LongRunHighGrayLevelEmphasis |  | p | p | p | p | p | p | p/d |  | p |  |
|  | LongRunLowGrayLevelEmphasis |  | p |  | p | p | p | p |  | p |  | p |
|  | LowGrayLevelRunEmphasis |  |  |  |  |  |  | p | p/d | p | p |  |
|  | RunEntropy | p/d | p/d | p | p/d | p/d | p/d | p | p/d |  | p/d/**△**/**√** | p |
|  | RunLengthNonUniformity | p |  | p |  |  |  | p | p | p | p | p |
|  | RunLengthNonUniformityNormalized |  | d |  | p/d | p/d | p/d |  |  |  |  |  |
|  | RunPercentage |  | p/d |  | p/d | p/d | p/d |  |  |  |  |  |
|  | RunVariance |  | p |  | p | p | p | p |  |  |  |  |
|  | ShortRunEmphasis |  | p/d |  | p/d | p/d | p/d |  |  |  |  |  |
|  | ShortRunHighGrayLevelEmphasis | p/d | p |  | p/d | p/d | p/d |  | p/d |  | p |  |
|  | ShortRunLowGrayLevelEmphasis |  | p/d |  | p/d | p/d | p/d |  | p/d |  |  |  |
| GLSZM | GrayLevelNonUniformity |  |  | p |  |  |  | p | p | p | p | p |
|  | GrayLevelNonUniformityNormalized | p |  |  |  |  |  |  | p/d |  | p |  |
|  | GrayLevelVariance | p |  |  |  |  |  |  | p/d |  |  |  |
|  | HighGrayLevelZoneEmphasis | d |  |  |  |  |  |  | p/d |  | p |  |
|  | LargeAreaEmphasis |  | p |  | p | p | p | p |  |  |  |  |
|  | LargeAreaHighGrayLevelEmphasis |  | p |  | p | p | p | p |  |  |  |  |
|  | LargeAreaLowGrayLevelEmphasis |  | p |  | p | p | p | p |  | p |  | p |
|  | LowGrayLevelZoneEmphasis |  |  |  |  |  |  |  | p/d |  |  |  |
|  | SizeZoneNonUniformity | p |  | p |  |  |  | p | p | p | p | p |
|  | SizeZoneNonUniformityNormalized | d |  |  |  |  |  | p/d |  |  |  |  |
|  | SmallAreaEmphasis |  |  |  | p/d | p/d | p/d | p/d | d |  | p |  |
|  | SmallAreaHighGrayLevelEmphasis | d |  |  | p/d | p/d | p/d | p/d/**△** | p/d |  | p |  |
|  | SmallAreaLowGrayLevelEmphasis |  |  | p | d | p/d | p/d | p/d/**√** |  |  |  |  |
|  | ZoneEntropy | p |  |  |  |  |  | p/d | p |  | p | p |
|  | ZonePercentage |  | p |  | p/d | p/d | p/d | p |  |  |  |  |
|  | ZoneVariance |  |  |  |  |  |  | p |  |  |  |  |
| NGTDM | Busyness |  |  |  |  |  |  | p |  | p |  |  |
|  | Coarseness |  |  | p/d |  |  |  | p/d/**△** |  | p/d | p | d |
|  | Complexity | p |  |  |  |  |  |  | p/d |  |  |  |
|  | Contrast |  |  |  |  |  |  |  |  |  |  |  |
|  | Strength |  |  |  |  |  |  | p/d |  | p |  |  |
| **total** | **post** | **36** | **27** | **14** | **31** | **37** | **37** | **39** | **51** | **13** | **36** | **14** |
|  | **delta** | **17** | **15** | **1** | **26** | **27** | **24** | **14** | **27** | **1** | **1** | **1** |

^*^ No radiomics features were extracted

^#^ p refers to the features retained by the Mann‒Whitney U test of the postgroup

d refers to the features retained by the Mann‒Whitney U test of delta-group

√ refers to the features retained by LASSO regression of the postgroup

**△** refers to the features retained by LASSO regression of the delta group.

| Parameter | Manufacturer | | | | | |
| --- | --- | --- | --- | --- | --- | --- |
|  | Siemens SOMATOM Force | Philips iCT 256 | TOSHIBA | GE MEDICAL SYSTEMS Revolution CT | NMS NeuViz Glory | MinFound ScintCareCT 128 |
| Tube voltage (kVp) | 120 | 120 | 120 | 100 | 120 | 120 |
| Tube current (mA) | 69-184 | 351-404 | 102-158 | 199-299 | 225-250 | 360 |
| Rotation time (s) | 0.5 | 0.5 | 0.5 | 0.5 | 0.5 | 0.5 |
| Single Collimation Width | 0.6 | 0.625 | 0.5 | 0.625 |  | 0.625 |
| Total Collimation Width | 57.6 | 80 | 32 | 80 |  | 40 |
| Slice Thickness | 1 | 1 | 1 | 0.625、1.25 | 1 | 1 |
| Reconstruction Diameter | 432 | 500 | 425.781 | 500 | 500 | 395.16 |
| Spiral Pitch Factor | 0.6 | 0.914 | 0.828 | 0.992188 | 0.8-0.9 | 1.25 |
| Convolution Kernel | Br36d\1 | B | FC08 | STANDARD | F10 | Abdomen_STND |
| Exposure Time | 500 | 547-548 | 500 | 500 | 2830-4720 | 5230 |
| Matrix | 512*512 | 512*512 | 512*512 | 512*512 | 512*512 | 512*512 |

TableS2: The detailed information on CT image acquisition.

| Feature | ICC2 |
| --- | --- |
| original_shape_Maximum2DDiameterSlice | 0.987486 |
| lbp-2D_gldm_DependenceNonUniformityNormalized | 0.995207 |
| lbp-3D-m2_firstorder_Kurtosis | 0.997891 |
| lbp-3D-m2_gldm_DependenceNonUniformityNormalized | 0.995207 |
| lbp-3D-k_glszm_SmallAreaLowGrayLevelEmphasis | 0.807308 |
| square_glrlm_RunEntropy | 0.997128 |

Table S3: ICCs of features in the postgroup

Table S4: ICCs of features in the deltagroup

| Feature | ICC3k |
| --- | --- |
| original_shape_Elongation | 0.963185 |
| original_shape_MinorAxisLength | 0.994743 |
| original_shape_SurfaceVolumeRatio | 0.993096 |
| lbp-3D-m2_firstorder_Kurtosis | 0.909243 |
| lbp-3D-k_glszm_SmallAreaHighGrayLevelEmphasis | 0.691275 |
| lbp-3D-k_ngtdm_Coarseness | 0.995108 |
| log-sigma-4-0-mm-3D_glcm_Autocorrelation | 0.859052 |
| square_glrlm_RunEntropy | 0.866436 |
